# Drought, Ash and Soil Legacies Shape Seed Germination Responses in *Pinus canariensis* C. Sm. & DC. Forests

**DOI:** 10.3390/plants15142242

**Published:** 2026-07-22

**Authors:** María A. Pérez-Fernández, Irene de Lara del Rey, Cristina González-Montelongo, José Ramón Arévalo

**Affiliations:** 1 Department of Physical, Chemical and Natural Systems, University Pablo de Olavide, Avenida Rectora Rosario Valpuesta, 1, 41089 Dos Hermanas, Sevilla, Spain; iiadedel@alu.upo.es; 2 Department of Botany, Ecology, and Plant Physiology, Faculty of Sciences, Universidad de La Laguna, 38206 San Cristóbal de La Laguna, Tenerife, Spainjarevalo@ull.edu.es (J.R.A.)

**Keywords:** fire ecology, seed dormancy, soil microorganisms

## Abstract

Fire plays a central role in shaping regeneration dynamics in *Pinus canariensis* C. Sm. & DC. forests, yet the relative importance of different fire-related cues and post-fire environmental filters remains poorly understood. We experimentally evaluated the effects of drought, heat, smoke, ash and soil microbial extracts on the germination of seven co-occurring species representing the main functional groups of these forests. Germination responses varied markedly among species, revealing that no single cue dominates early recruitment. Drought emerged as the strongest and most consistent filter, sharply reducing germination success and slowing emergence in *P. canariensis* and *Fabaceae*, while *Poaceae* remained largely insensitive under the imposed water potentials. Ash enhanced germination in *P. canariensis* and *Cynosurus echinatus* L., indicating that nutrient-rich post-fire substrates can facilitate emergence when moisture is available. Soil and microbial extracts accelerated germination in fast-emerging grasses, providing the first experimental evidence that post-fire soil legacies influence recruitment in Macaronesian pine forests. In contrast, heat acted as a species-specific cue, with only limited stimulation at moderate temperatures and widespread declines under extreme heat, while the smoke treatment used in this study produced negligible effects across all species. Across treatments, germination timing emerged as a key functional axis, structuring regeneration niches, with rapid-germinating *Poaceae* favoured under increasing aridity. Our findings highlight the dominant role of hydric stress, the selective relevance of ash and soil legacies, and the functional divergence among species, offering new mechanistic insights into post-fire regeneration under current and future climate scenarios.

## 1. Introduction

Fire is a major ecological driver in many Mediterranean-type ecosystems, where seeds are exposed to a suite of environmental changes—including heat pulses, smoke-derived compounds, ash deposition, altered soil chemistry, and shifts in microbial communities—that can act as cues for germination or, alternatively, impose constraints on seedling establishment [1]. These fire-related signals have been widely documented as modulators of seed dormancy and germination in numerous plant families, particularly in fire-prone regions [2,3].

Heat shock is one of the most widely studied fire cues. Short exposures to high temperatures can break physical dormancy in species with hard seed coats, especially among *Fabaceae* and some *Poaceae*, while excessive temperatures may reduce viability [4,5,6]. Smoke contains bioactive molecules such as karrikins that stimulate germination in many species from fire-adapted ecosystems, although responses are highly species-specific [7,8,9]. Ash deposition can modify soil pH, nutrient availability, and osmotic conditions, thereby influencing germination either positively or negatively, depending on species traits and ash concentration [10,11]. Despite these well-established mechanisms, the combined or contrasting effects of heat, smoke, and ash on co-occurring species within the same community remain insufficiently explored.

Fire also induces profound changes in soil microbial communities. Heating of the upper soil layers, deposition of pyrogenic compounds, and post-fire nutrient pulses can reduce microbial biomass and diversity, alter functional groups, and shift plant–microbe interactions [10,12]. Soil microorganisms can influence germination by producing hormones, enzymes, or allelopathic compounds [13], yet their role as post-fire germination cues remains one of the least understood aspects of fire ecology.

In addition to fire-derived factors, post-fire drought is a recurrent feature of Mediterranean-type climates. Reduced water availability can strongly limit germination and early seedling survival, acting as a selective filter immediately after fire [14,15]. Although drought is not a fire cue in the strict sense, it is a recurrent post-fire environmental stressor that strongly interacts with heat, smoke, ash and soil changes. By limiting water availability during imbibition and emergence, drought can override or modify the effects of fire-derived cues and therefore plays a key role in shaping species’ regeneration niches.

The Canary Islands host extensive forests dominated by *Pinus canariensis*, an endemic pine with a unique combination of fire-adaptive traits, including thick bark, epicormic re-sprouting, and serotinous cones [16]. While the fire ecology of *P. canariensis* has been documented, much less is known about the germination responses of its understory species—particularly *Fabaceae* and *Poaceae*—that contribute to post-fire community assembly. Understanding how these species respond to multiple fire-related cues and to drought is essential for predicting regeneration patterns in these insular ecosystems [17,18].

Here, we examine the germination responses of *P. canariensis* and six common understory species to five key environmental factors associated with fire or post-fire conditions: heat shock, smoke, ash, soil microbial extracts from burnt and unburnt soils, and drought. By comparing species from different functional groups, we aim to determine to what extent fire-derived cues and water availability shape regeneration dynamics in *P. canariensis* forests.

To frame this study within current fire ecology theory, we tested the following mechanistic hypotheses: (i) brief exposure to high temperatures acts as a signal capable of breaking dormancy or activating germination in species adapted to fire regimes; (ii) smoke- and ash-derived compounds function as chemical cues that modulate germination responses in species sensitive to post-fire environments; (iii) shifts in soil microbial communities following fire alter the biological and chemical context experienced by seeds, thereby influencing germination outcomes; (iv) reduced water availability after fire acts as an environmental filter that limits germination in species sensitive to hydric stress. These hypotheses aim to provide a mechanistic understanding of how multiple fire-derived and post-fire factors interact to structure early regeneration dynamics in *P. canariensis* ecosystems.

## 2. Results

### 2.1. Germination Under Drought Stress

**Figure 1:**
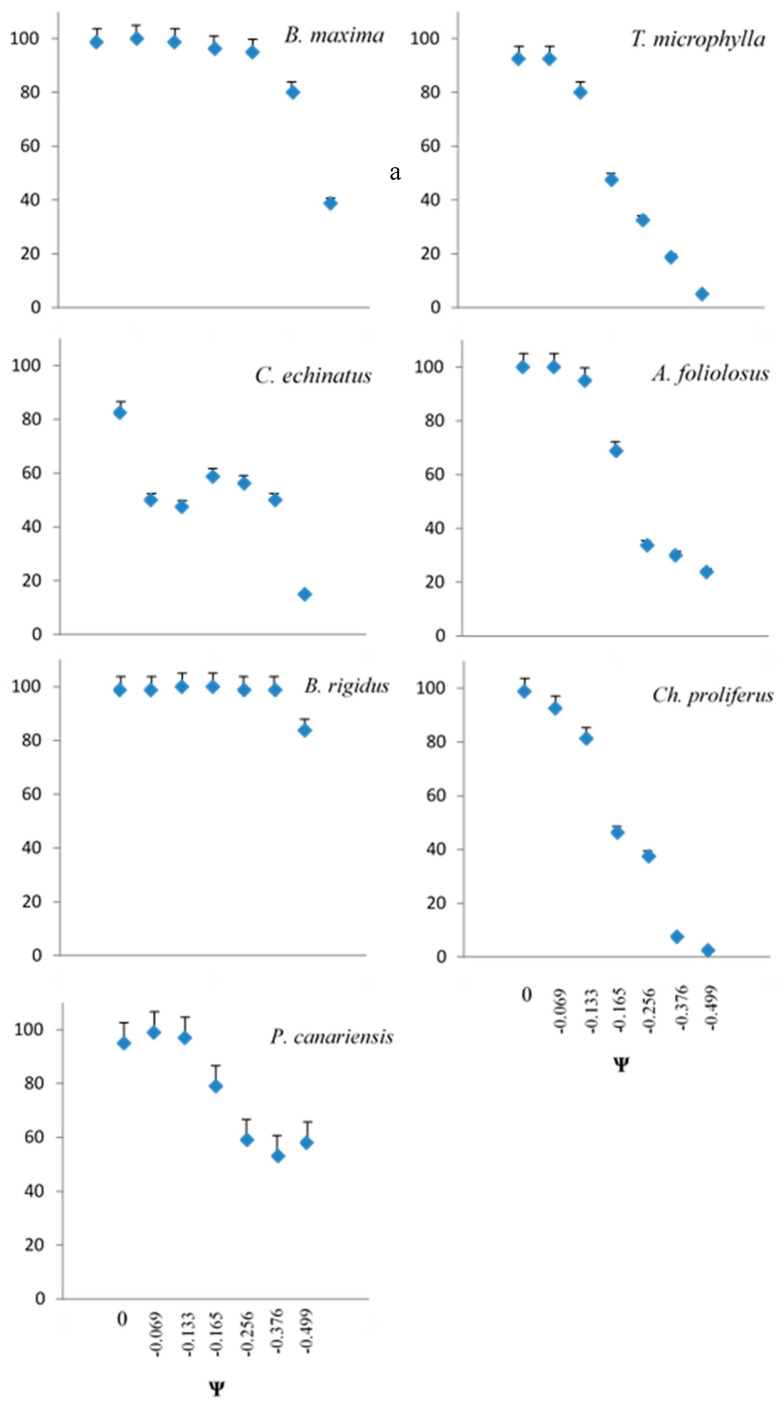
Final germination percentages (±sd) of seven species from a *Pinus canariensis* forest along a PEG-induced drought gradient. Water potentials (0 to −0.50 MPa) and corresponding PEG concentrations (0, 80, 111, 124, 154, 184 and 215 g/L) were calculated at 22 °C following Equation (1). Columns with different letters were significantly different for each species (Tukey’s HSD, *p* < 0.05).

Germination responses across the drought gradient showed clear non-linear and species-specific patterns (Figure 1). *Pinus canariensis* and the three Fabaceae species maintained high germination at the highest water potentials tested, followed by a marked decline as water availability decreased. In contrast, the three *Poaceae* species showed weak or inconsistent responses to water potential. In some cases, such as *Bromus rigidus* Roth and *Cynosurus echinatus* L., germination was relatively low under control conditions but increased under moderate water stress before declining again at lower potentials. A similar pattern has been reported in other species and may reflect a stimulatory effect of mild stress on germination processes. Overall, these results indicate that germination responses to drought are not linear, but depend on species-specific thresholds and sensitivities.

Across the drought gradient, germination dynamics mirrored the decline in germination percentage. In the three *Poaceae* species, TI and MTG showed relatively small variation across the gradient, with no significant differences detected among several treatments (Figure 1). In *Fabaceae*, TI decreased significantly with increasing drought stress, while MTG increased significantly, whereas in *Poaceae*, no significant differences were detected among treatments (Figure 1). *P. canariensis* showed the strongest temporal response: as water potential decreased, TI dropped sharply and MTG increased markedly, revealing a pronounced slowdown in germination speed under drought. Overall, species with the steepest declines in germination percentage also showed the largest increases in MTG and reductions in TI (Table 1).

### 2.2. Heat Treatment

**Figure 2:**
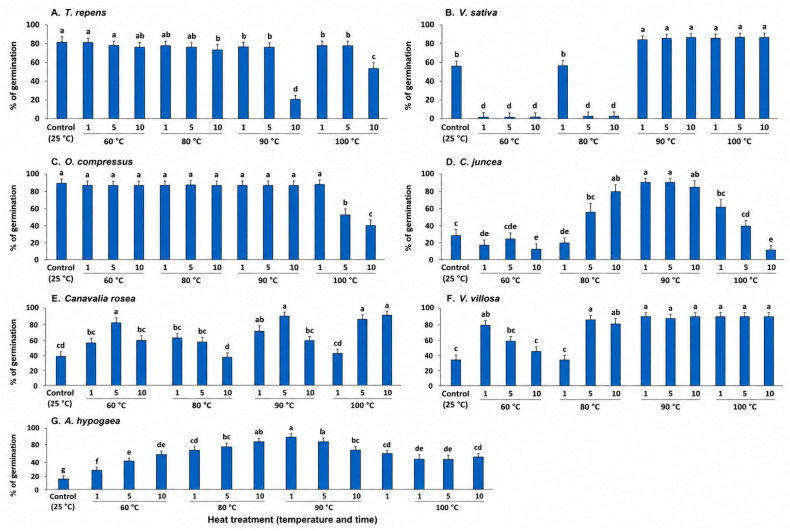
Germination response of *B. maxima*, *C. echinatus*, *B. rigidus*, *T. microphylla*, *A. foliolosus*, *C. proliferus*, and *P. canariensis* seeds after heat shock treatments of different intensity and exposure time. Within each species (graph), columns with different letters were significantly different for each species (Tukey’s HSD, *p* < 0.05).

Exposure to high temperatures significantly increased germination of *P. canariensis* relative to the control, although not all temperature-time combinations differed significantly (Figure 2). Maximum germination occurred at 100 °C for 5–10 min and at 120 °C for 1 min, with these treatments showing significantly higher values than other combinations (Figure 2). Temperatures above 100 °C generally produced higher germination than the control, although not all differences among treatments were statistically significant (Figure 2). In *Poaceae*, *Briza maxima* and *Bromus rigidus* showed similar responses: both species failed to germinate at 120 °C and 150 °C for both 5 and 10 min. *Cynosurus echinatus* exhibited increased germination in 13 of the 21 heat treatments, with the highest values at 60 °C for 10 min and 150 °C for 1 min.

All three *Fabaceae* species showed significantly higher germination at temperatures ≥100 °C across exposure times. *Adenocarpus foliolosus* (Aiton) DC. and *Teline microphylla* (DC.) P. E. Gibbs & Dingwall germinated poorly at the lowest temperatures, whereas *Chamaecytisus proliferus* (L. f.) Link displayed a more uniform response, with maximum germination at 120 °C and minimum germination at 100 °C for 5 min (Figure 2).

No significant interaction between temperature and exposure time was detected. Heat treatments produced clear species-specific shifts in germination timing. In *Poaceae*, TI remained relatively stable at moderate temperatures, but MTG increased substantially at the highest temperatures and longest exposures, indicating delayed germination even when final percentages were not strongly affected. *Fabaceae* showed more pronounced temporal sensitivity: TI decreased and MTG increased under high temperature pulses, especially at 100–130 °C, where germination was both reduced and delayed. *T. microphylla* and *C. proliferus* were particularly sensitive, showing sharp increases in MTG at high temperatures. *P. canariensis* maintained relatively stable TI and MTG at moderate heat but exhibited slower germination (higher MTG) at the most extreme treatments. These patterns confirm that heat affects not only germination success but also the speed and synchrony of germination (Table 1).

### 2.3. Smoke Treatment

**Figure 3:**
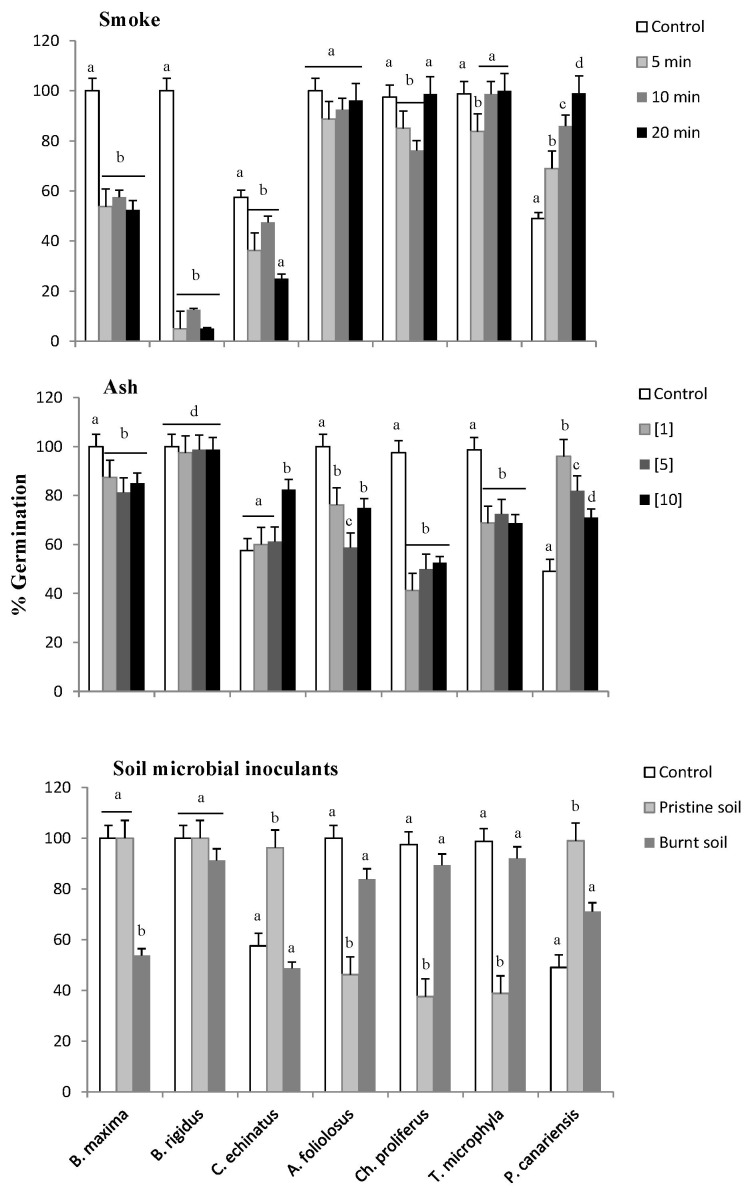
Final percentages of germination after 35 days of *B. maxima*, *C. echinatus*, *B. rigidus*, *T. microphylla*, *A. foliolosus*, *C. proliferus*, and *P. canariensis* seeds treated with pulses of smoke (5, 10 or 20 min) and three concentrations of ash in solution (1, 5 or 10 g/liter). Columns with different letters were significantly different for each species (Tukey’s HSD *p* < 0.05).

Smoke exposure increased germination of *P. canariensis* at all durations tested (Figure 3). In *Poaceae*, smoke significantly reduced germination in the three species. In *Fabaceae*, smoke had no significant effect except for a reduction in *C. proliferus* at 5 and 10 min and in *T. microphylla* at 5 min. Germination responses did not differ significantly among exposure durations.

Smoke exposure generally accelerated germination in species that responded positively in percentage terms. In *Fabaceae*, smoke-stimulated species showed higher TI and lower MTG, indicating faster and more synchronous germination. *Poaceae* species showed weaker responses: TI increased slightly in *C. echinatus*, while MTG decreased modestly, consistent with their mild response to smoke. *P. canariensis* showed a clear temporal response, with higher TI and lower MTG at effective smoke exposures, matching its increased germination percentage. Overall, smoke tended to enhance germination speed in the same species where it increased germination success (Table 1).

### 2.4. Ash Treatment

Ash solutions increased germination of *P. canariensis*, with the highest germination at 1 g L^−1^, followed by 5 and 10 g L^−1^ (Figure 3). In contrast, none of the *Fabaceae* species showed a clear stimulatory response to ash, with germination remaining similar to the control across concentrations. Ash did not significantly affect germination in *Poaceae* species, although *C. echinatus* showed significantly higher germination at 10 g L^−1^.

Ash treatments produced distinct temporal responses across species. In *P. canariensis*, all ash concentrations increased TI and reduced MTG, indicating faster germination consistent with its higher germination percentages. In *Poaceae*, only *C. echinatus* showed a temporal response, with higher TI and lower MTG at 10 g L^−1^, matching its stimulation in germination percentage. *B. maxima* and *B. rigidus* showed no meaningful changes in TI or MTG, consistent with their lack of response in germination percentage. *Fabaceae* species showed stable TI and MTG across concentrations, reflecting the absence of stimulatory effects. Thus, ash influenced germination timing only in species where it increased germination success, although these effects were not always associated with statistically significant differences among treatments (Table 1).

### 2.5. Soil Microbial Extracts

Microbial extracts from unburnt soils increased the level of germination in *P. canariensis* and *C. echinatus* (Figure 3). Extracts from burnt soils also increased germination level and rate in *P. canariensis,* although differences between treatments were not always statistically significant (Table 1). In *Fabaceae*, extracts from unburnt soils significantly reduced germination level, onset, and rate in all three species. Extracts from burnt soils did not show significant differences relative to the control.

Differences between pristine and burnt soil extracts were also reflected in germination timing. Species showing higher germination in burnt soil extracts—particularly *Fabaceae*—also exhibited higher TI and lower MTG, indicating faster and more synchronous germination. *Poaceae* species showed minimal temporal responses, with TI and MTG remaining similar across extracts, consistent with their weak or absent stimulation. *P. canariensis* displayed moderate increases in TI and reductions in MTG in burnt soil extracts, matching its improved germination. Overall, temporal patterns reinforced the species-specific responses observed in germination percentages (Table 1).

**Figure 4:**
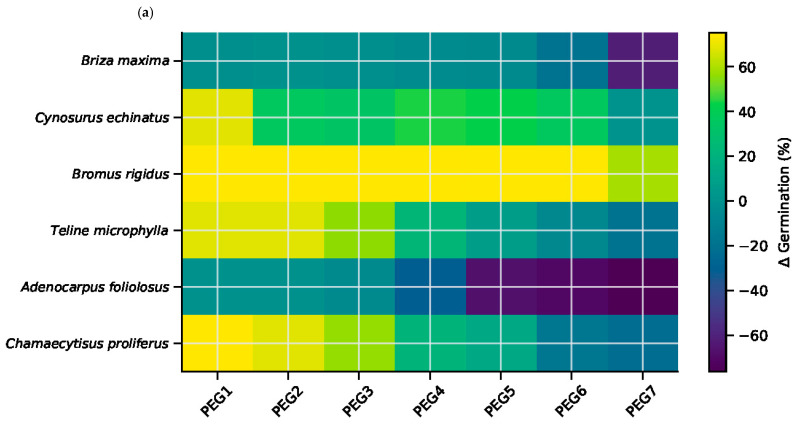

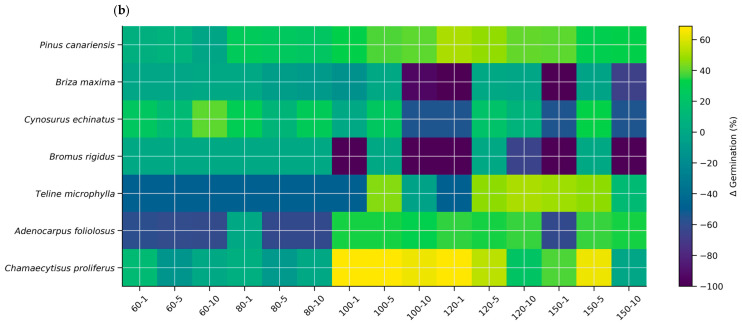
Heatmaps illustrating the magnitude of germination responses (Δ germination percentage relative to control conditions) across experimental treatments. (**a**) Responses to drought (D), smoke (S), and ash (A) treatments. (**b**) Responses to heat treatments across temperature (°C)–exposure time (min) combinations. Heat treatments are identified as temperature (°C)–exposure time (min); for example, 80–10 denotes exposure to 80 °C for 10 min. Effect sizes were calculated as the difference in germination percentage between each treatment and its corresponding control. Warmer colors indicate positive responses (stimulation of germination), whereas cooler colors indicate negative responses (inhibition). Ψ values correspond exactly to those reported in Table 2.

The magnitude and direction of treatment effects varied markedly among species and treatments (Figure 4). Strong positive and negative responses were observed across the drought gradient, whereas smoke and ash treatments produced more variable and species-specific effects. Heat treatments showed pronounced responses depending on both temperature and exposure time, highlighting the importance of thermal thresholds in germination responses.

**Table 2:** Amounts of PEG added per liter of distilled water to create water potential gradients under which germination of *Briza maxima* [1], *Bromus rigidus* [2], *Cynosorus echinatus* [3], *Adenocarpus foliolosus* [4], *Chamaecytisus proliferus* [5], and *Teline microphylla* [6] were tested, as well as grams of PEG per gram of H_2_O.

	PEG g/L	g PEG/g H_2_O	Ψ
[1]	79.6	0.0796	−0.0686
[2]	111	0.1109	−0.1334
[3]	123.5	0.1235	−0.1652
[4]	154	0.1539	−0.2564
[5]	184	0.1840	−0.3673
[6]	214.5	0.2145	−0.4988

## 3. Discussion

Seed responses to fire-related cues are central to regeneration in fire-prone ecosystems, and our results reveal substantial interspecific variation among species from *P. canariensis* forests. This diversity aligns with the idea that fire acts as a strong evolutionary filter shaping seed traits, dormancy mechanisms and germination syndromes [19,20]. No single cue strongly constrained germination responses; instead, early recruitment arose from the interaction of multiple fire-derived and post-fire factors, each acting with different strength depending on species traits and functional identity [2].

Among all treatments, drought produced the clearest and most consistent pattern. Germination of *P. canariensis* and *Fabaceae* declined sharply with decreasing water potential, whereas *Poaceae* remained largely insensitive. Although germination in Poaceae showed only modest declines within the tested drought range, this pattern should not be interpreted as complete insensitivity, but rather as a comparatively weak response relative to the other functional groups. This contrast is consistent with well-established ecological theory in Mediterranean-type ecosystems, where water limitation acts as a strong environmental filter shaping early recruitment [2,21,22]. Similar patterns have been reported in other Mediterranean systems, where germination can decline more than 50% under moderate water stress, particularly in species with larger seeds and higher metabolic demands [14,15]. In the Canary pine forest, this filter is particularly strong because post-fire soils experience intense evaporative demand, reduced canopy shading and rapid surface drying [23,24,25]. The slowdown in germination speed indicated by higher MTG and lower TI in drought-sensitive species reinforces the idea that hydric stress not only reduces germination success but also delays emergence, thereby narrowing the temporal window for establishment before summer drought [21,26,27]. These patterns support the view that post-fire drought acts as a key constraint on recruitment by both limiting germination and filtering species according to their physiological tolerance to water deficit [24]. Functional differences among species help explain these patterns. *Fabaceae* and *P. canariensis* produce relatively large seeds with higher metabolic demands during imbibition, making them more vulnerable to water deficits, whereas *Poaceae* species, with small seeds and rapid germination strategies, are adapted to exploit short moisture pulses typical of post-fire microsites [28,29]. These contrasting strategies are consistent with trait-based frameworks linking seed size and germination speed to drought sensitivity and resource use in Mediterranean-type and subtropical fire-prone ecosystems [21,22,23,24,25,26,27,28].

Although *P. canariensis* has evolved thick bark, epicormic resprouting and serotinous cones [17,23], our results suggest that its regeneration niche is strongly constrained by post-fire drought—an increasingly relevant limitation under current climatic trends [30]. This apparent mismatch between adult fire resistance and early life-stage sensitivity highlights the importance of considering regeneration bottlenecks when evaluating species responses to fire and climate change. These results are consistent with the view of fire as an intrinsic component of the dynamics of these ecosystems, and suggest that its management should aim to maintain fire regimes as close as possible to natural conditions [17].

Heat pulses are widely recognized as important germination cues in fire-prone ecosystems [8,31,32]. However, in *P. canariensis* forests, heat acted as a selective and relatively weak cue. Moderate temperatures produced limited stimulation, while extreme temperatures reduced germination percentage and speed across most species. Similar response patterns have been reported in other fire-prone systems, where germination responses to heat are constrained within a narrow thermal threshold and rapidly decline beyond species-specific limits [8,33,34]. These responses are consistent with physiological mechanisms underlying dormancy release: temperatures slightly above ambient can promote imbibition in hard-seeded species, while higher temperatures can damage embryos or disrupt membrane integrity, thereby limiting germination success [8,33,34].

Species-specific responses reinforce this interpretation. *P. canariensis* showed weak stimulation under moderate heat and declines under extreme heat, consistent with its evolutionary strategy: seeds are protected inside serotinous cones and typically experience relatively mild heating during crown fires [5,35]. *Fabaceae* showed only weak stimulation at intermediate temperatures and declines at higher ones, suggesting relatively narrow thermal thresholds that might be exceeded during high-intensity fires in Canary pine forests [36]. *Poaceae* were largely insensitive, reflecting their reliance on moisture pulses rather than thermal cues [20,35]. Importantly, heat responses were strongly modulated by drought: even when heat slightly increased germination, subsequent hydric stress often reduced or offset these effects. This interaction highlights the importance of considering multiple, interacting drivers when interpreting germination responses in post-fire environments.

Smoke-derived compounds such as karrikinolide stimulate germination in many fire-prone floras [7,37], but across the seven species studied, smoke produced only weak and inconsistent responses. This pattern is consistent with the relatively low smoke sensitivity reported for Mediterranean-type and subtropical floras [38,39] and may also reflect the fire regime of *P. canariensis* forests, where high-intensity crown fires generate rapid smoke dispersion and limited penetration into the understory [24]. Across all functional groups, *P. canariensis*, *Fabaceae* and *Poaceae* showed negligible or highly variable responses, suggesting that smoke is not a consistently effective post-fire germination cue in this system. The weak response to smoke parallels the limited stimulation observed under heat and further emphasizes the strong influence of drought and soil-mediated processes as key drivers of germination patterns. The weak response to smoke observed here may reflect the intrinsic insensitivity of the study species, but it may also be influenced by variation in smoke chemistry. Natural smoke contains karrikins and other germination-active compounds, yet their concentration varies among fuel types, combustion conditions and exposure duration. Because our smoke was generated from local vegetation without chemical characterization, we cannot fully separate species-level insensitivity from potential differences in compound abundance. This interpretation is presented as a plausible ecological explanation rather than a demonstrated mechanism, consistent with the limits of the experimental design. Future work, including chemical analyses of smoke solutions, would help clarify these mechanisms. Future studies should also investigate how different dormancy-breaking mechanisms, including physical dormancy in Fabaceae, interact with fire-related cues to influence post-fire recruitment. Ash produced clearer and more consistent effects than smoke or heat. *P. canariensis* responded positively across ash concentrations, suggesting that ash-induced increases in pH or nutrient availability may facilitate germination, consistent with field observations of seedling establishment in ash-rich microsites [30]. *Cynosurus echinatus* also responded positively at high ash concentrations, reflecting its capacity to exploit nutrient-rich, disturbed substrates. *Fabaceae* showed no clear response to ash, possibly due to limited seed coat permeability, whereas most *Poaceae* species remained largely insensitive. Ash stimulation was strongest in species performing well under favorable moisture, suggesting that ash acts as a facilitating cue, but mainly when drought does not impose strong constraints on germination. These results indicate that the effects of ash are highly context-dependent and interact with water availability to shape germination outcomes.

Soil microbial extracts revealed the influence of post-fire soil legacies, including changes in nutrient availability, allelopathic compounds and microbial communities. Fire can alter soil microbial composition, reduce fungal biomass, stimulate pyrophilous bacteria and modify nutrient cycling [40,41]. However, very few studies have tested the direct effects of soil or microbial extracts on germination, and none in Macaronesian ecosystems. Our results therefore provide novel evidence that burnt soil extracts can increase germination in *P. canariensis*, while reducing germination in *C. echinatus* and *B. maxima*, with no significant effect on *B. rigidus*. These contrasting responses suggest that post-fire soil chemistry and microbial shifts may differentially influence germination among species, potentially promoting rapid emergence in opportunistic colonizers while constraining others. *P. canariensis* showed modest acceleration, while *Fabaceae* exhibited variable responses. Soil-mediated processes appear particularly important for species relying on post-fire resource pulses, and likely interact with ash effects and drought sensitivity to shape germination patterns under post-fire conditions [42].

Germination timing emerged as a central axis structuring post-fire regeneration. *Poaceae* consistently germinated rapidly across treatments, including ash and soil extracts, and maintained relatively high performance under moderate drought. This rapid emergence strategy allows grasses to exploit short moisture pulses immediately after fire [43,44]. In contrast, *Fabaceae* exhibited slower and more drought-sensitive germination, restricting recruitment to relatively infrequent moist periods. *P. canariensis* showed intermediate timing, with drought-induced delays indicating dependence on short moist intervals that are becoming less frequent under current climate change [21]. The interplay between timing, drought sensitivity, and responsiveness to fire-derived cues suggests a potential mechanism contributing to species coexistence under the same fire regime.

Taken together, our results indicate that post-fire regeneration in *P. canariensis* forests arises from the interaction of multiple fire-derived cues and strong post-fire environmental filters, rather than from any single dominant mechanism. Although controlled laboratory conditions do not replicate the full complexity of field environments, the breadth of our experimental approach allows identifying the main drivers of germination responses under fire-related cues. Drought consistently emerged as a major constraint, influencing the effects of heat, smoke, ash and soil legacies, and interacting with species-specific traits to shape patterns of germination success and timing. The contrasting responses among *P. canariensis*, *Fabaceae* and *Poaceae* point to the coexistence of distinct regeneration niches within the same fire regime, ranging from drought-sensitive, slow-germinating species to opportunistic grasses capable of rapid emergence under fluctuating moisture.

Regional climate projections for the Canary Islands indicate a clear trend towards warmer and drier conditions during the 21st century, with mean temperature increases of ~2–4 °C by 2070–2100 under high-emission scenarios and a marked rise in drought and aridity indices, particularly at mid-elevations where *P. canariensis* forests are concentrated [14,41]. These changes are expected to intensify post-fire hydric stress and shorten the duration of moist windows available for germination and early establishment.

As aridity intensifies across the Canary Islands, these functional differences are likely to become increasingly important in shaping post-fire community trajectories. Incorporating hydric constraints, soil-mediated processes and germination timing into predictive frameworks will therefore improve our understanding of regeneration dynamics in Macaronesian pine forests under future climate and fire scenarios. Although this study captures key germination responses under controlled conditions, it does not encompass the full complexity of field microsites or post-fire processes such as predation, which may further influence recruitment outcomes. Future work integrating field-based experiments and longer-term demographic data will be necessary to refine these interpretations.

From a management perspective, our results suggest that post-fire regeneration in *P. canariensis* forests may depend strongly on the availability of short moisture windows immediately after fire. Practices that preserve soil structure, minimize erosion and retain ash-rich microsites could facilitate recruitment of drought-sensitive species such as *P. canariensis* and *Fabaceae*. Because early establishment is constrained by hydric stress, post-fire interventions that reduce surface drying—such as maintaining coarse woody debris, limiting soil disturbance or avoiding immediate salvage logging—may help sustain suitable microsites for germination. In contrast, opportunistic *Poaceae* are likely to dominate under increasingly arid conditions, potentially altering post-fire trajectories. These patterns highlight the need to integrate drought sensitivity and soil-mediated processes into fire management planning, particularly under projected increases in temperature and aridity in the Canary Islands.

## 4. Conclusions

Post-fire regeneration in *Pinus canariensis* forests emerges from the interaction of multiple fire-derived cues and strong post-fire environmental filters, rather than from any single dominant mechanism. Drought consistently acted as the main constraint, reducing germination success and delaying emergence in *P. canariensis* and *Fabaceae*, while Poaceae maintained relatively high performance under moderate water stress. Heat and smoke produced only weak or inconsistent stimulation, whereas ash and burnt-soil extracts exerted context-dependent effects that were strongest under favorable moisture conditions.

These contrasting responses reveal distinct regeneration niches within the same fire regime: drought-sensitive, slow-germinating species such as *P. canariensis* and *Fabaceae*, and opportunistic grasses capable of rapid emergence under fluctuating moisture. As aridity intensifies across the Canary Islands, these functional differences are likely to become increasingly important in shaping post-fire community trajectories. Incorporating hydric constraints, soil-mediated processes and germination timing into predictive frameworks will improve our understanding of regeneration dynamics in Macaronesian pine forests under future climate and fire scenarios.

## 5. Materials and Methods

### 5.1. Study Species and Seed Collection

**Figure 5:**
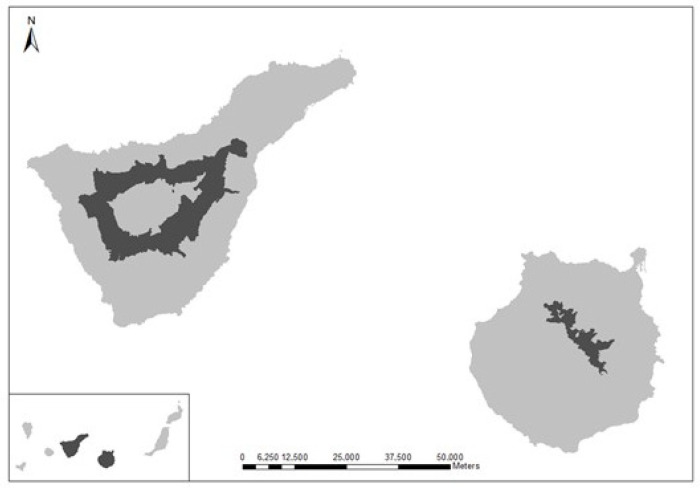
Sampling sites Tenerife ((**left**)—Natural Park Corona Forestal in dark) and Gran Canaria ((**right**)—natural Reserve Los Marteles in dark) in the Canary Islands (Spain) from where seeds of *Briza maxima*, *Bromus rigidus*, *Cynosorus echinatus*, *Adenocarpus foliolosus*, *Chamaecytisus proliferus* and *Teline microphylla* were collected.

We examined the germination responses of seven species characteristic of *Pinus canariensis* forests: three *Poaceae* (*Briza maxima*, *Bromus rigidus*, and *Cynosurus echinatus*), three *Fabaceae* (*Adenocarpus foliolosus*, *Chamaecytisus proliferus*, and *Teline microphylla*), and *P. canariensis*. These species were selected because they are among the most abundant under the canopy of *P. canariensis,* are strongly associated with fire-prone environments, and were the only species for which sufficient quantities of seeds could be collected to ensure adequate replication in the germination experiments. In addition, the selected species represent the dominant functional groups involved in post-fire recruitment in these forests, including grasses, legumes and the canopy-forming species. For each species, seed mass, viability, life form and dispersal period were obtained from batches of 100 seeds per species (Table 3). Seeds were collected during their natural dispersal period in late summer from two protected areas: Corona Forestal (Tenerife) and Los Marteles (Gran Canaria), at 1400–1700 m a.s.l. (Figure 5). These two sites encompass the elevational and structural variability of *P. canariensis* forests and are widely used as reference stands in ecological and regeneration studies [24,45]. Seeds were stored dry at 10 ± 1 °C and 20% relative humidity following [46] guidelines. Seeds of all species were collected independently on both islands (Tenerife and Gran Canaria). To ensure adequate sample sizes and to maximize representativeness across the distribution range of *P. canariensis* forests, seeds from both provenances were pooled for the germination assays.

### 5.2. General Germination Procedures

Prior to treatments, *P. canariensis* seeds were cold-stratified for 15 days at 2 °C in moist sand. This short stratification period enhances embryo hydration and synchronizes germination without inducing secondary dormancy [23]. Cold stratification followed the recommendations of the International Seed Testing Association [46] and published germination protocols for *P. canariensis*. Seeds of the *Poaceae* and *Fabaceae* families received no pretreatment. These species do not require cold stratification under standard germination protocols, and species-specific pretreatments were applied only when necessary to obtain ecologically representative germination responses rather than as experimental treatments. All seeds were surface-sterilized (with 70% ethanol for 5 min followed by 1% sodium hypochlorite for 3 min) and rinsed thoroughly. Germination tests were conducted in 9-cm sterile Petri dishes lined with Whatman No. 2 filter paper and moistened with the appropriate solution (distilled water, PEG, ash, or microbial extract). Seeds exposed to the smoke treatment were subsequently incubated on filter paper moistened with distilled water. Dishes were incubated under a 10 h dark/14 h light thermoperiod (18 ± 1 °C/22 ± 1 °C). These conditions reflect mean spring temperatures in mid-elevation Canary pine forests and are commonly used in germination studies from the region [23]. Germination (radicle emergence) was recorded daily for 35 days. Non-germinated seeds were dissected to assess viability. Each treatment consisted of five replicates of 20 seeds each.

### 5.3. Simulation of Drought Stress

Drought was simulated using polyethylene glycol (PEG 6000) solutions adjusted to seven water potentials (Table 2). The selected water potentials (0 to −0.5 MPa) encompass the range typically experienced in post-fire soils during the first weeks after burning in Mediterranean and Macaronesian ecosystems [14,47,48,49]. PEG concentrations were calculated following the temperature-dependent equation of [50]:

Ψ = 0.130 [PEG]^2^ T − 13.7 [PEG]^2^ (1)

where Ψ is the osmotic potential (MPa), PEG is the concentration expressed as grams of PEG per gram of water, and *T* is the temperature (°C). All calculations were performed for 22 °C, the optimal germination temperature for the study species. Filter papers were replaced periodically to maintain a stable water potential throughout the experiment. PEG solutions were renewed every 48 h, following [51], to prevent osmotic drift and ensure a consistent water potential.

### 5.4. Heat Treatment

To simulate heat pulses associated with fire, seeds were exposed to combinations of seven temperatures (60, 80, 90, 100, 120, 130, and 150 °C) and three exposure times (1, 5, and 10 min) in a calibrated dry-air oven. Temperature and exposure times were selected to reflect realistic soil heating conditions during wildfires. According to [52], heat pulses at 2–3 cm depth typically last between 1 and 15 min and reach temperatures ranging from 44 to 150 °C. Temperatures above 100 °C occur at 1–2 cm depth during high-intensity crown fires, whereas pulses of 60–90 °C are typical of surface fires and smouldering phases [20,32]. Temperature inside the oven was continuously monitored using an internal calibrated thermocouple placed at seed level, which reached the target temperature within 30–40 s and remained stable throughout the exposure period (±1 °C). This ensured that all seeds experienced the intended thermal pulse for the exact duration of each treatment. After treatment, seeds were transferred immediately to Petri dishes and incubated under standard conditions.

### 5.5. Smoke Treatment

Smoke exposure followed a modified [53] protocol. Seeds were placed in a 250-mL vacuum flask into which smoke was continuously funneled for 5, 10, or 20 min. Smoke was generated from a mixture of dry and green plant material representative of the study area. This mixture produces a smoke composition comparable to that generated during natural fires in Canary pine forests [24]. Exposure times were selected to span the range known to stimulate germination in Mediterranean species [53]. The temperature inside the flask remained at 21 ± 1 °C, and the smoke pH was monitored (pH 4.8). Seeds were then incubated under standard conditions [54].

### 5.6. Ash Treatment

White ash was produced by burning leaves and branches of *P. canariensis* and the three *Fabaceae* species under open-air conditions. Equal amounts of dry plant material were placed in a 50 × 50 cm metal container and ignited using a butane flame applied to the finest dry fractions. Combustion proceeded freely under ambient oxygen supply and was completed within approximately 35 min. The ash was allowed to cool to room temperature and collected. This procedure was repeated three times, and all ash batches were homogenized to obtain a representative composite sample.

Ash samples were homogenized and their chemical properties were determined in an aqueous extract (1:20 ash:water) following the AOAC International procedures [55]. After 5 min of mechanical shaking, the suspension was filtered through 0.45 µm membranes. pH and electrical conductivity were measured with a Crison GLP 22 (Crison Instruments, S.A., Barcelona, Spain electrode. Ammonium, nitrate, sulfate and phosphate concentrations were quantified by UV–VIS spectrophotometry using the Nessler method, in a microplate reader, against standard calibration curves (0–1 ppm for NH_4_^+^, NO_3_^−^ and PO_4_^3−^; 0–40 ppm for SO_4_^2−^) [55,56]. Ash was suspended in distilled water to obtain solutions of 1, 5, and 10 g L^−1^. Each Petri dish received 10 mL of the corresponding solution. The pH of the suspensions ranged from 7.7 to 8.1 [54]. Data on ash nutrient content are given as Supplementary Materials (Table S1).

### 5.7. Soil Microbial Extracts

Soil microbial suspensions were prepared from the top 10 cm of unburnt soil and from soil collected two weeks after a wildfire in a *P. canariensis* stand. For each extract, 200 g of soil was mixed with 500 mL of sterile water, stirred for 1 h, allowed to settle, and the supernatant was filtered through 1 µm paper to remove coarse particles and fungal hyphae while retaining bacteria and soluble metabolites. The resulting suspension was not sterilized, as the aim was to preserve the active water-soluble microbial and chemical fraction known to respond rapidly to fire, including pyrophilous bacteria and heat-tolerant saprotrophs [56,57].

To distinguish microbial metabolic effects from the chemical solute fraction of the soil, we included a sterilized-soil control. For this control, an aliquot of the unburnt-soil suspension was autoclaved at 121 °C for 20 min and cooled to room temperature. This treatment eliminates microbial activity while preserving the water-soluble chemical fraction of the soil. Seeds in the sterilized-soil treatment received 10 mL of this suspension, applied with the same frequency as in the non-sterilized treatments.

To verify microbial viability, aliquots of each extract were plated on LB and tryptophan agar, and colony-forming units (CFUs) were recorded after incubation. Seeds were watered exclusively with the original, non-sterilized extract, and each Petri dish received 10 mL of either unburnt-soil or burnt-soil suspension. Plates were visually monitored throughout the experiment to ensure microbial activity remained within ecologically realistic levels without excessive overgrowth.

### 5.8. Germination Metrics

Germination percentage was calculated after adjusting for non-viable seeds. Germination rate was quantified using a modified Timson’s index. This index is widely used in fire-related germination studies because it integrates both speed and uniformity of emergence [58,59]:

(2) $$Rate\;of\;germination\;=\frac{⅀G}{t}$$

where G is the germination percentage recorded every two days and t is the total duration of the germination test. Higher values of this index indicate faster germination.

We also calculated the mean time to germination (MTG), which reflects the speed at which seeds initiate germination [4]:

(3) $$\mathrm{MTG}=\frac{\sum (n_{i}\cdot d_{i})}{N}$$

where n_i_ is the number of seeds germinated on day i, d_i_ is the incubation day, and N is the total number of germinated seeds. Lower MTG values indicate quicker germination.

All indices were calculated using daily germination counts over the 35-day incubation period.

### 5.9. Statistical Analyses

Germination percentages were arcsine-square-root transformed, and assumptions of normality and homoscedasticity were checked using Levene’s test. For drought, smoke, and ash treatments, differences among water potentials, smoke-exposure durations, and ash concentrations were evaluated using one-way ANOVA, followed by Tukey’s HSD when significant effects were detected. Heat treatments were analysed using two-way ANOVA, with temperature and exposure time as fixed factors, including their interaction, and Tukey’s HSD for post hoc comparisons. Interaction terms were tested but were not significant, and therefore are not discussed further. For microbial extracts, differences between unburnt and burnt soil extracts were assessed using one-way ANOVA for each species. Statistical significance was set at *p* < 0.05, and all analyses were performed in SPSS 22.0. ANOVA was selected because germination responses showed homogeneous variances after transformation and because treatments were fully crossed and balanced, making linear models appropriate for detecting treatment effects.

In addition to significance testing, the magnitude of treatment effects was evaluated by calculating effect sizes as the change in germination percentage (Δ%) relative to control conditions for each species and treatment. These values were used to generate heatmaps representing both the direction and strength of treatment responses across the drought gradient. The heatmaps were produced using Python (3.10.12 Python Software Foundation, Wilmington, DE, USA) with the matplotlib 3.8.2 and NumPy 1.26.4 libraries, providing a graphical representation complementary to the statistical analyses.

## Figures and Tables

**Table 1:** Values of Timson’s index(TI) and mean time to germination (MTG) of seeds of seven species from a *Pinus canariensis* forest, under several treatments (*n* = 100 seeds per species, data are average and SD). Asterisks indicate significant differences with the control (*p* < 0.05). Asterisks next to the numbers indicate significant differences after Tukey HSD. TI is dimensionless; MTG is expressed as the number of days to germination.

	Species	*B. maxima*		*B. rigidus*		*C. echinatus*		*A. foliolosus*		*C. proliferus*		*T. microphylla*		*P. canariensis*	
Treatment		TI	MTG	TI	MTG	TI	MTG	TI	MTG	TI	MTG	TI	MTG	TI	MTG
Control		3.010	1.756	3.025	1.563	2.442	10.972	4.023	4.963	3.002	3.854	2.992	4.997	2.431	11.000
60 ºC	1 min	3.250	1.367	3.333	1.338	2.625	10.810	3.208	4.519	3.208	3.577	3.183	4.634	2.835	10.668
	5 min	3.292	2.127	3.333	1.325	2.000	11.218	3.042	4.387	3.254	3.962	3.333	3.701	2.968	10.804
	10 min	3.333	2.025	3.250	1.557	3.167	13.553	3.252	3.564	3.002	3.240	3.250	3.449	2.145	10.026
80 ºC	1 min	3.333	2.075	3.333	1.525	2.833	8.821 *	3.208	3.351	2.792	5.313 *	3.167	4.145	3.021	6.587
	5 min	3.083	3.658	3.333	1.575	2.167	10.731	3.250	3.722	2.000	5.063	2.125	4.880	2.981	6.247
	10 min	3.042	3.342	3.333	1.701	2.583	12.348	0	0	1.708	1.667	3.250	4.423	2.887	5.996
90 ºC	1 min	3.208	2.013	3.333	1.525	2.583	7.839 *	3.250	4.481	3.125	5.373	0	0	3.145	5.012
	5 min	3.292	2.295	3.333	1.550	1.583	12.289	3.208	3.416	2.542	5.231	3.292	3.962	3.247	5.114
	10 min	3.000	7.944 *	3.292	3.127 *	2.792	13.941	0.042	1.00*	1.583	1.026	0	0	3.254	4.998 *
100 ºC	1 min	3.333	4.663 *	3.333	2.63 *	2.625	12.181	0	0	3.167	2.638	3.125	3.147	4.526	3.148 *
	5 min	2.792	6.062 *	3.333	2.25 *	1.752	11.659	0	0	0.333 *	1.503	1.500	4.167	5.026 *	3.014 *
	10 min	2.833	4.074 *	0	0	1.875	6.159 *	3.125	4.597	0.625 *	2.600	0	0	5.102 *	3.069 *
120 ºC	1 min	3.250	4.000	3.292	1.759	2.708	14.446	0.125	12.241	2.667	5.922	0	0	4.965 *	3.985 *
	5 min	0.208	10.000	0	0	0	0	0.083	7.559	1.510	3.125	0	0	4.769 *	4.128 *
	10 min	0	0	0	0	0	0	0.042	9.014	1.292	2.818	0	0	3.905	4.255 *
130 ºC	1 min	3.292	4.291 *	3.292	1.013	2.542	9.672	2.125	4.039	2.500	5.25	0	0	3.625	4.961 *
	5 min	3.042	6.221 *	1.125 *	4.259 *	2.083	7.275 *	0	0	1.208	2.621	0	0	3.756	4.877 *
	10 min	0	0	0	0	0	0	0	0	0	0	0	0	3.596	5.267 *
150 ºC	1 min	3.250	3.551	3.333	1.213	2.958	11.268	0.042 *	5.000	3.083	4.635	0	0	2.998	7.554
	5 min	1.125 *	10.185 *	0	0	0	0	0	0	0.125 *	3.333	0	0	3.751	7.633
	10 min	0	0	0	0	0	0	0	0	0	0	0	0	4.965 *	7.968
Smoke	5 min	2.159	1.896	0.698	5.933	1.943	13.259	3.659	5.011	2.769	5.588	2.908	5.669	4.998 *	5.852
	10 min	2.083	1.599	1.001	6.057	2.081	12.907	3.478	5.106	2.635	5.732	2.915	4.896	5.758 *	3.829 *
	20 min	2.402	3.058	0.861	6.117	1.147	12.555	3.625	5.556	3.594	4.803	3.008	5.057	5.887 *	2.361 *
Ash	1 g/L	3.108	2.458	2.395	1.776	2.509	11.055	2.033	7.336	1.105	5.966	1.158	7.896	5.769 *	3.463 *
	5 g/L	2.903	2.411	2.996	1.854	2.183	10.590	1.769	7.055	0.961 *	6.752	1.069	7.299	3.751	4.092 *
	10 g/L	2.611	2.599	3.010	1.866	3.115	8.633	1.821	7.598	0.893 *	6.884	1.114	8.011	4.965 *	4.956 *
Soil	Pristine	3.330	5.225	3.330	2.038	3.208	7.247	1.542	5.351	1.250	5.933	1.292	5.355	5.903	6.733
Extract	Burnt	1.542	4.488	2.858	2.849	1.625	3.718	2.708	10.299	2.093	9.240	2.292	6.550	4.965	3.836 *

**Table 3:** Attributes of the species used in this study.

Species	Botanical Family	Life Form	Dispersal Period	Natural Distribution	Seed Mass (g)	Seed Viability (%)
*Briza maxima*	Poaceae	Annual	March to May	Croplands, pasturelands, paths	2.8	100
*Bromus rigidus*	Poaceae	Annual	May to August	Croplands, pasturelands, paths	1.23	95
*Cynosurus echinatus*	Poaceae	Annual	March to August	Croplands and open fields	1.83	100
*Adenocarpus foliolosus*	Fabaceae	Shrub or small tree	February to August	Open fields	5.7	100
*Chamaecytisus proliferus*	Fabaceae	Shrub or small tree	May to August	Highlands, drylands	18.57	100
*Teline microphylla*	Fabaceae	Shrub or small tree	May to August	Highlands, drylands	7.57	100
*Pinus canariensis*	Pinaceae	Tree	After fires	Hihlands	17.3	99

## Data Availability

Data will be made available upon request.

## Supplementary Materials

The following supporting information can be downloaded at https://www.mdpi.com/article/10.3390/plants15142242/s1.
