# Supplementary material for: Drought, Ash and Soil Legacies Shape Seed Germination Responses in Pinus canariensis C. Sm. & DC. Forests
Source: Plants (Basel). 2026 Jul 22;15(14):2242. doi: 10.3390/plants15142242 (PMC13416483; doi:10.3390/plants15142242)
Supplement: Supplementary file 1 [file plants-15-02242-s001.zip › plants-4404362-supplementary.pdf]

Table S1. Mean values (± standard error of the mean) of pH, electrical conductivity (EC), and concentrations of chemical species in the aqueous extracts obtained from ash produced by burning *P. canariensis* understory vegetation. N= 4.

**Variable**	**Ash**
pH	8.1 ± 0.3
**CE (µS cm-1)**	123.61 ±18.21
**N-NO_3 (mg kg-1)**	5.14 ± 2.6
**S-SO_4^{-2} (mg kg-1)**	763.54 ± 103.7
**P (mg kg-1)**	22.05 ± 2.8
